# Rapid Cerebrospinal Fluid Dipstick Testing for Early Diagnosis of Acute Bacterial Meningitis in Children: A Prospective Observational Study in Nepal

**DOI:** 10.7759/cureus.88738

**Published:** 2025-07-25

**Authors:** Biplav Ghimire, Ram H Chapagain, Ganesh K Rai, Arika Poudel, Ajit Rayamajhi

**Affiliations:** 1 Department of Pediatrics, Kanti Children's Hospital, Kathmandu, NPL; 2 Department of Pediatrics, National Academy of Medical Sciences, Kathmandu, NPL; 3 Department of Pediatrics, Kathmandu Medical College Teaching Hospital, Kathmandu, NPL; 4 Department of Ophthalmology, B.P. Koirala Lions Center for Ophthalmic Studies, Institute of Medicine, Kathmandu, NPL

**Keywords:** acute bacterial meningitis, cerebrospinal fluid, dipstick test, pediatric diagnostics, resource-limited settings

## Abstract

Introduction

Acute bacterial meningitis (ABM) is a life-threatening emergency requiring prompt diagnosis and treatment. In resource-limited settings, rapid and reliable diagnostic methods are essential. This study evaluates the diagnostic accuracy of a urinary reagent dipstick test applied to cerebrospinal fluid (CSF) samples in children suspected of having ABM.

Methods

A prospective observational study was conducted at Kanti Children’s Hospital, Kathmandu, Nepal, from February 2019 to April 2021. Children aged between three months and 14 years undergoing lumbar puncture for suspected meningitis were included. CSF samples were tested using Combur-10 (Roche Diagnostics GmbH, Mannheim, Germany) urinary dipsticks for leukocytes and protein and compared to standard laboratory analyses. Diagnostic accuracy was measured using sensitivity, specificity, predictive values, and Cohen’s kappa coefficient.

Results

Seventy-six children were enrolled, with a median age of 4.5 years, and 48 (63%) participants were males. Laboratory analysis identified ABM in 21 cases (28%) based on the World Health Organization (WHO) criteria for probable bacterial meningitis. Dipstick testing diagnosed 26 cases (34.2%). The dipstick demonstrated a sensitivity of 95% (95% CI: 78.4% - 99.3%) and a specificity of 89% (95% CI: 77.8% - 95.1%). The positive predictive value (PPV) was 77% (95% CI: 57.5% - 89.4%), the negative predictive value (NPV) was 98% (95% CI: 87.3% - 99.8%), and the overall diagnostic accuracy was 91% (95% CI: 81.2% - 96%). Agreement between dipstick and conventional methods was statistically significant (Cohen’s kappa = 0.785, p = 0.001).

Conclusions

Rapid dipstick testing of CSF is a reliable, cost-effective tool for early ABM diagnosis in children. It is particularly useful in settings lacking advanced laboratory infrastructure.

## Introduction

Meningitis is an inflammation of the meninges, most commonly caused by bacterial or viral infections of the cerebrospinal fluid (CSF) [1]. In neonates, frequent bacterial pathogens include Group B *Streptococcus*, *Escherichia coli*, and *Listeria* monocytogenes, while in older children, *Haemophilus influenzae* type B, *Neisseria meningitidis*, and *Streptococcus pneumoniae* are more common. Viral, fungal, and parasitic causes are also recognized [1].

The clinical features of acute bacterial meningitis (ABM) include fever, altered mental status, neck stiffness, signs of raised intracranial pressure, seizures, and focal neurological deficits [2]. Prompt diagnosis is essential, as complications such as cranial nerve palsies, hydrocephalus, hearing loss, and cognitive impairment may occur, usually due to delayed treatment [2]. Worldwide, mortality remains less than 10% beyond the neonatal period, but long-term sequelae are seen in up to 50% of survivors [3]. In Nepal, the incidence of ABM in children is estimated to be approximately 100 per 100,000 children annually, with mortality rates ranging from 7.7% to 33.3%, depending on treatment availability [4-6].

CSF analysis remains the diagnostic gold standard, involving microscopy, culture, and sensitivity testing [2]. However, results can take hours to days, and many rural and primary health centers lack the infrastructure for timely diagnosis. This delays treatment and increases complications and referral burdens. A delay in the administration of antibiotics is associated with a significant increase in the risk of mortality and neurological sequelae [7,8].

This study investigates the use of the Combur-10 (Roche Diagnostics GmbH, Mannheim, Germany) urinary dipstick on CSF as a rapid, point-of-care diagnostic tool, which has not been reported previously from Nepal. The sensitivity, specificity, positive predictive value (PPV), negative predictive value (NPV), and accuracy of the dipstick tests were evaluated in comparison with conventional laboratory CSF analysis and microscopy. If proven reliable, this approach could facilitate earlier diagnosis and treatment, especially in low-resource settings, potentially reducing complications and improving outcomes.

## Materials and methods

Study design and setting

This was a hospital-based observational study conducted at Kanti Children’s Hospital, Maharajgunj, Kathmandu, Nepal, from February 2019 to April 2021.

Study population

The study included children aged >3 months to ≤14 years, admitted to any hospital units (emergency, observation, medical, paying, special cabin, pediatric intensive care unit, or pediatric intermediate care unit) with suspected ABM who underwent lumbar puncture and met inclusion criteria. Exclusion criteria included contraindications for lumbar puncture, ventriculoperitoneal shunt infections, prior antibiotic use, traumatic CSF samples, or lack of consent. These were excluded from the study due to the possibility of unreliable clinical presentation and CSF pictures.

Neonates and infants up to the age of three months were excluded from the study, as both term and preterm infants with meningitis can have nonspecific presentations. Moreover, in neonates, even without meningitis, CSF leukocyte count can be high and CSF protein values variable, leading to difficulty in diagnosing meningitis via the standard set for our study [9,10]. Moreover, CSF protein levels in the absence of meningitis are variable in this population [10]. Similarly, the upper limit of age of the patients for the study was taken as 14 years, as patients above 14 years of age are not admitted to Kanti Children's Hospital.

Sample size

The sample size was calculated using the following formula:

$n = \frac{z^2pq}{d^2}$

where z = 1.96 (95% CI), p = accuracy (%), q = 100 - p, and d = 7.5% of p.

Using prior data from Kumar A et al., p = 94.63% for leukocytes and 90.67% for protein/glucose, yielding sample sizes of 39 and 71, respectively [11]. Thus, a minimum of 71 participants was required.

Definitions

Due to the low CSF culture positivity rates in Nepal (ranging from 4.4% to 7.2%) [6,12], the World Health Organization (WHO) criteria for probable bacterial meningitis were applied at the time of the study. According to this criterion, probable bacterial meningitis was defined as clinically suspected meningitis plus CSF findings of either a turbid appearance or a leukocyte count >100 cells/µL or a leukocyte count between 10 and 100 cells/µL combined with either a protein level >100 mg/dL or a glucose level <40 mg/dL [13].

ABM was clinically suspected in patients with fever (>38.5°C rectal or >38.0°C axillary) plus altered consciousness, or signs of meningeal irritation (neck stiffness, Kernig's sign, Brudzinski's sign), or features of increased intracranial pressure (headache, vomiting, papilledema, cranial nerve palsies, hypertonia, hyperreflexia, extensor plantar reflexes, seizures, hypertension, bradycardia), or focal neurological deficits [3, 13].

Dipstick testing

The Combur-10 urinary dipstick was used for rapid testing of CSF. The container holding the dipsticks was stored at room temperature and promptly recapped after each dipstick was removed, in accordance with the manufacturer’s instructions. CSF samples were collected using sterile technique into sterile vials, following standard lumbar puncture protocols. Dipstick testing was performed immediately after sample collection to minimize the risk of false-positive or false-negative results due to contamination or delayed analysis. The CSF dipstick interpretation was conducted by a single investigator. For quality control, every tenth sample was retested and independently interpreted by a second investigator. As a result, no additional training of hospital staff was necessary for this procedure. Interpretation thresholds are mentioned in Table 1 [14].

**Table 1:** Interpretation thresholds for cerebrospinal fluid parameters with the Combur-10 dipstick test

Parameters	Interpretation
Leukocytes 1+	10–75/µL
Leukocytes 2+	75–500/µL
Leukocytes 3+	>500/µL
Protein 1+	30–100 mg/dL
Protein 2+	100–500 mg/dL
Protein 3+	>500 mg/dL
Glucose negative	<2.8 mmol/L (50 mg/dL)
Glucose positive	≥2.8 mmol/L

CSF glucose could not be included in our evaluation due to a difference in the value detectable by dipstick (<2.8 mmol/L or 50 mg/dL) and the reference value used in the WHO definition (<40 mg/dL) [13, 14].

Sampling technique

Convenience sampling was employed. All eligible children who underwent lumbar puncture and met the inclusion criteria were enrolled.

Data collection

Whenever a lumbar puncture was planned by the concerned physicians in the above-mentioned hospital units, the study team was informed. After confirming eligibility, informed consent and assent (as applicable) were obtained. A structured clinical assessment was performed. CSF samples were collected in three parts: routine analysis (appearance, glucose, protein, and leukocytes); Gram stain and culture; and dipstick testing (performed by the investigator).

The investigator responsible for dipstick testing and interpretation was blinded to the laboratory results, and laboratory personnel were similarly blinded to the dipstick findings. In the laboratory, CSF leukocyte counts were performed manually using a Neubauer chamber. Protein and glucose concentrations were measured using standard colorimetric assays, with routine quality control procedures conducted daily and weekly.

Statistical analysis and data management

The collected data were entered into Microsoft Excel, version 10 (Microsoft Corp., Redmond, WA), followed by proper data cleaning procedures. The cleaned data were then transferred to Statistical Package for the Social Sciences (SPSS) software, version 16 (SPSS Inc., Chicago, IL) for analysis. Sensitivity, specificity, PPV, NPV, and overall accuracy of the dipstick test were calculated for leukocytes, protein, and for the diagnosis of ABM. Cohen’s weighted kappa test was performed to assess agreement between the dipstick test results and standard laboratory findings.

Patient confidentiality was strictly maintained by using only hospital or inpatient ID numbers for identification. The dataset was password-protected and accessible only to the principal investigator, co-investigator, and research guide. All data will be securely destroyed five years after the completion of this study.

Ethical considerations

Ethical approval was obtained from the Institutional Review Board of the National Academy of Medical Sciences (NAMS) (Ref No. 554/076/077) on January 6, 2020. Written informed consent was taken from guardians, and assent from children when applicable. Participation was voluntary, and confidentiality was maintained throughout.

## Results

Patient characteristics

A total of 76 pediatric patients were included in the study. Their demographic and clinical characteristics are summarized in Table 2. Of the patients, 48 (63%) were male, with a male-to-female ratio of 1.7:1. Thirty-seven patients (49%) were older than five years. Fever was the most common presenting symptom, observed in 71 patients (93%), followed by altered sensorium in 46 (60%). Meningeal signs were present only in 15 patients (20%), while signs of raised intracranial pressure (ICP) were noted in nine (12%).

**Table 2:** Patient characteristics and presenting features ICP: intracranial pressure

Patient characteristic	Frequency/Value (%)
Sex	
Male	48 (63%)
Female	28 (37%)
Median age	54 months (4 years and 6 months)
Number of patients aged	
<1 year	16 (21%)
1–5 years	23 (30%)
>5 years	37 (49%)
Symptoms and signs	
Fever	71 (93%)
Altered sensorium	46 (60%)
Seizure	42 (55%)
Meningeal signs	15 (20%)
Features of raised ICP	9 (12%)
Focal neurological deficits	15 (20%)
Blood total leukocyte count (TLC), median	11,000/µL

CSF analysis by conventional laboratory method

All CSF samples were studied for gross appearance, protein, leukocyte count, glucose, Gram stain, and culture. The median and range of CSF leukocytes, protein, and glucose, along with other CSF parameters, are listed in Table 3.

**Table 3:** CSF parameters (conventional laboratory analysis) CSF: cerebrospinal fluid; min: minimum; max: maximum

CSF parameters	Results
CSF appearance	Clear (all patients)
Gram stain positive	1 patient (Gram-negative bacilli)
CSF culture positive	1 patient (*Escherichia coli*)
CSF laboratory parameters	Median (Min, Max)
Protein	63 mg/dl (16, 597)
Leukocyte count	0 /µL (0, 750)
Glucose	57.5 mg/dl (7, 100)

Based on the WHO criteria for probable bacterial meningitis, 21 patients (28%) were diagnosed with acute bacterial meningitis. Of these, 15 were male and six were female (male-to-female ratio: 2.5:1). Their ages ranged from three months to 13 years, with a mean age of 5.98 ± 3.39 years, a median of six years, and an interquartile range of 12 to 120 months. 

CSF analysis by rapid dipstick

On analysis by rapid dipstick, CSF for leukocytes was found to be negative in 48 (63%), 1+ in 21 (28%), 2+ in six (8%), and 3+ in one (1%) sample. Similarly, CSF for protein was found to be negative in 11 (14%), 1+ in 37 (49%), 2+ in 26 (34%), and 3+ in two (3%) samples. Each finding of the rapid dipstick was compared with the corresponding findings by conventional laboratory analysis. Dipstick findings matched with CSF findings in 65 cases (86%) for protein and 72 cases (95%) for CSF leukocytes. CSF findings for leukocytes and protein after analysis by rapid dipstick and corresponding laboratory findings are summarized in Tables 4, 5.

**Table 4:** Number of cases according to dipstick and lab values for CSF leukocytes CSF: cerebrospinal fluid

Rapid dipstick test for CSF leukocytes	Laboratory value for CSF leukocytes (/µL)			
	<10	10-75	75-500	>500
Negative	48	0	0	0
1+	3	17	1	0
2+	0	0	6	0
3+	0	0	0	1

**Table 5:** Number of cases according to dipstick and lab values for CSF protein CSF: cerebrospinal fluid

Rapid dipstick test for CSF protein	Laboratory value for CSF protein (mg/dl)			
	<30	30-100	100-500	>500
Negative	6	5	0	0
1+	2	35	0	0
2+	1	2	23	0
3+	0	0	1	1

CSF findings after analysis by rapid dipstick were compared with the standard of this study (WHO criteria [13]), and a diagnosis of acute bacterial meningitis was made in 26 patients (34%).

The sensitivity of the rapid dipstick test in the diagnosis of ABM in comparison to the conventional laboratory analysis was found to be 95% (95% CI: 78.4% - 99.3%). Similarly, specificity was 89% (95% CI: 77.8% - 95.1%), PPV was 77% (95% CI: 57.5% - 89.4%), NPV was 98% (95% CI: 87.3% - 99.8%), and accuracy was 91% (95% CI: 81.2% - 96%), respectively. Cohen's weighted kappa was calculated and found to be 0.785, with a standard error of 0.076, which was statistically significant with a p-value of 0.001. These calculations were based on values in Table 6.

**Table 6:** Comparison of diagnosis of ABM by rapid dipstick test with the conventional laboratory test ABM: acute bacterial meningitis

ABM by dipstick	ABM by conventional laboratory analysis		
	ABM	No ABM	Total
ABM	20	6	26
No ABM	1	49	50
Total	21	55	76

Individual component of the findings of rapid dipstick analysis

Each finding of the rapid dipstick was compared with the corresponding finding by conventional laboratory analysis, and calculations were done accordingly. These calculations were not possible for CSF protein 3+ and CSF leukocytes 3+. 

CSF Protein by Dipstick

Finding of CSF protein as negative by dipstick had a sensitivity of 67%, specificity of 93%, PPV of 55%, NPV of 95%, and an accuracy of 89% in comparison with laboratory findings. Similarly, protein 1+ had a sensitivity of 83%, a specificity of 94%, a PPV of 95%, an NPV of 82%, and an accuracy of 88%. CSF protein 2+ had improved sensitivity and NPV of 96% and 98%, respectively, with 94% specificity, 88% PPV, and 95% accuracy. 

CSF Leukocytes by Dipstick

CSF leukocytes negative by dipstick had an excellent sensitivity and specificity of 95% and 100%, respectively, with PPV, NPV, and accuracy at 100%, 89%, and 96%. CSF leukocytes as 1+ had a sensitivity of 100%, specificity of 93%, PPV of 81%, NPV of 100%, and an accuracy of 95%. Similarly, 2+ CSF leukocytes had a slightly lower sensitivity of 86% but showed better specificity and PPV of 100%, with NPV and accuracy at 99%.

## Discussion

ABM is a medical emergency requiring early diagnosis and treatment, often before the causative pathogen is identified [1]. The choice of initial antimicrobial therapy is based on the most common pathogen prevalent in a particular geographical area, its antibiotic sensitivity pattern, and the age group affected [1]. While advanced diagnostic methods like polymerase chain reaction (PCR) and latex agglutination exist for early and rapid diagnosis, they are costly and require skilled personnel, limiting their use in rural settings [1]. Our hospital-based prospective study at Kanti Children’s Hospital, Kathmandu, Nepal, evaluated the rapid urinary dipstick test against conventional CSF analysis for diagnosing ABM in 76 children.

The overall male-to-female ratio was 1.7:1, increasing to 2.5:1 among confirmed ABM cases. This trend is consistent with other studies, such as those by Pandey et al. (1.3:1) [15], Bhat et al. (1.5:1) [16], and Joshi et al. (2:1) [17], suggesting a potential gender-related susceptibility or disparities in healthcare access. Meningeal signs were present in only 15 patients (20%), substantially lower than the 60% reported by Zhang et al. [18] and 92% by Abdelmotaleb et al. [19]. This discrepancy may be attributed to the inclusion of a significant proportion (16 patients, 21%) of infants under one year of age in our study, as meningeal signs are often difficult to elicit in younger children. Notably, these signs typically develop after 12 months of age and may be absent altogether in children younger than 18 months [9]. The CSF culture positivity rate was low at 1.3%, in line with other studies from Nepal [6,12], but lower than rates reported internationally [11,20]. This could reflect prior antibiotic use and limited diagnostic resources. ABM was confirmed by laboratory findings in 21 patients (28%) in our study, closely matching the 26% reported by Romanelli et al. [21], though still lower than in some international studies [11,20], likely due to institutional diagnostic capacity constraints.

There have been prior studies evaluating the use of urine dipstick tests for rapid diagnosis of ABM [11,17,19,21,22]; however, our extensive literature review did not identify reports of its implementation in Nepal. Most studies have reported sensitivity, specificity, NPV, and PPV above 85%, which is similar to our study [11,17,19,21]. Different findings were reported by Maclennan et al., who found such rapid dipstick tests to be only 33% sensitive and 83% specific [22]. which might be due to subjective variations or differences in the epidemiology of bacterial meningitis and less developed laboratory facilities. Our study also showed dipstick protein 2+ or >100 mg/dl to have better accuracy than lower protein levels. In contrast to negative dipstick protein, having a lower sensitivity of 67%, negative dipstick leukocytes had excellent sensitivity of 94%, suggesting its possible use as a screening tool. Dipstick leukocytes 2+ or 100-500/µL showed specificity and PPV of 100% and could be a potential diagnostic tool. A meta-analysis of 13 studies reported similar findings of sensitivity and specificity of 92% and 98%, respectively, for dipstick leukocytes [14]. Given that each container holds 100 dipsticks, the cost of a rapid dipstick test was substantially lower than both minimalist and comprehensive CSF analyses for diagnosing ABM in resource-limited settings [23,24]. Thus, our findings support the dipstick test as a rapid, cost-effective, and accurate tool for early ABM diagnosis in resource-limited settings, where culture facilities are often inadequate.

While our findings are encouraging, some limitations may affect the generalizability of this study to the broader population of Nepal. The study was conducted at a single tertiary care center over a short duration, with a modest sample size and convenience sampling. Neonates and infants under three months, an age group with a high burden of ABM, were excluded due to differences in clinical presentation and laboratory parameters, limiting applicability. Other etiologies of meningitis, such as viral and fungal causes, were not assessed due to the unavailability of accurate confirmatory tests in low-resource settings.

Despite our best efforts, the study may not fully capture the true burden or characteristics of bacterial meningitis. The absence of randomization and a control group, necessitated by the observational nature of the study, a limited number of lumbar punctures, and the short study period, further constrain interpretation. The presence of false negatives and a few false positives underscores the need for confirmatory testing, which, although not widely available, remains essential for accurate diagnosis. CSF culture, the diagnostic gold standard, was not used as the reference due to its historically low yield in our setting. Additionally, CSF glucose was excluded from analysis because of threshold inconsistencies. While inter-observer variability was not a limitation in this study, future larger, multicenter studies will require standardized training to ensure consistency and reliability.

## Conclusions

Our findings indicate that the Combur-10 urinary dipstick test, when applied to CSF, may serve as a rapid, reliable, and cost-effective tool for the early diagnosis of acute bacterial meningitis in children, particularly in resource-limited settings. With high sensitivity and specificity, it offers a practical diagnostic tool in settings where access to advanced laboratory facilities is limited. Its simplicity and ease of use make it suitable for use in peripheral health centers and emergency settings, allowing the timely initiation of treatment and potentially reducing complications and mortality.

While our findings support the utility of dipstick testing, the study’s single-center design and modest sample size warrant further validation. Future multi-center studies with larger cohorts, inclusion of neonates, incorporation of CSF glucose analysis, and long-term follow-up are essential to strengthen and expand upon these findings. If validated, dipstick testing could be integrated into clinical protocols and national guidelines, improving early diagnosis and outcomes in pediatric acute bacterial meningitis, particularly in resource-limited environments.
